# Alterations in β-Cell Sphingolipid Profile Associated with ER Stress and iPLA_2_β: Another Contributor to β-Cell Apoptosis in Type 1 Diabetes

**DOI:** 10.3390/molecules26216361

**Published:** 2021-10-21

**Authors:** Tomader Ali, Xiaoyong Lei, Suzanne E. Barbour, Akio Koizumi, Charles E. Chalfant, Sasanka Ramanadham

**Affiliations:** 1 Research Department, Imperial College London Diabetes Center, Abu Dhabi 51133, United Arab Emirates; tomader.ali@gmail.com; 2 Department of Cell, Developmental, and Integrative Biology and Comprehensive Diabetes Center, University of Alabama at Birmingham, Birmingham, AL 35294, USA; xlei@uab.edu; 3 Department of Biochemistry and Biophysics, University of North Carolina at Chapel Hill, Chapel Hill, NC 27599, USA; barbours@email.unc.edu; 4 Department of Health and Environmental Sciences, Kyoto Graduate School of Medicine, Kyoto 606-8501, Japan; koizumi@kyoto-hokenkai.or.jp; 5 Department of Cell Biology, Microbiology and Molecular Biology, University of South Florida, Tampa, FL 33620, USA; cechalfant@usf.edu

**Keywords:** phospholipase A_2_, sphingolipids, diabetes, apoptosis

## Abstract

Type 1 diabetes (T1D) development, in part, is due to ER stress-induced β-cell apoptosis. Activation of the Ca^2+^-independent phospholipase A_2_ beta (iPLA_2_β) leads to the generation of pro-inflammatory eicosanoids, which contribute to β-cell death and T1D. ER stress induces iPLA_2_β-mediated generation of pro-apoptotic ceramides via neutral sphingomyelinase (NSMase). To gain a better understanding of the impact of iPLA_2_β on sphingolipids (SLs), we characterized their profile in β-cells undergoing ER stress. ESI/MS/MS analyses followed by ANOVA/Student’s *t*-test were used to assess differences in sphingolipids molecular species in Vector (V) control and iPLA_2_β-overexpressing (OE) INS-1 and Akita (AK, spontaneous model of ER stress) and WT-littermate (AK-WT) β-cells. As expected, iPLA_2_β induction was greater in the OE and AK cells in comparison with V and WT cells. We report here that ER stress led to elevations in pro-apoptotic and decreases in pro-survival sphingolipids and that the inactivation of iPLA_2_β restores the sphingolipid species toward those that promote cell survival. In view of our recent finding that the SL profile in macrophages—the initiators of autoimmune responses leading to T1D—is not significantly altered during T1D development, we posit that the iPLA_2_β-mediated shift in the β-cell sphingolipid profile is an important contributor to β-cell death associated with T1D.

## 1. Introduction

Diabetes is a consequence of pancreatic islet β-cell dysfunction and/or reduced peripheral insulin sensitivity, and both type 1 and type 2 diabetes (T1D and T2D) are associated with β-cell apoptosis [1,2]. Therefore, it is important to elucidate the underlying involved mechanisms so that treatment regimens can be developed to protect the β-cells and prevent or delay diabetes development.

In addition to the extrinsic (receptor-mediated) and intrinsic (mitochondrial) apoptotic pathways, ER stress is recognized as an inducer of apoptosis in several disease states, including diabetes [3]. Reports from both experimental models and clinical settings have linked diabetes development with ER stress-induced β-cell apoptosis [4], and studies in our laboratory revealed an important role for the group VIA Ca^2+^-independent phospholipase A_2_ (iPLA_2_β) in this process [5]. The iPLA_2_s belong to a family of PLA_2_s that include the secretory and cytosolic PLA_2_s [6]. All of the PLA_2_s catalyze the hydrolysis of the *sn*-2 substituent from membrane glycerophospholipids [7] to release a free fatty acid and a lysophospholipid. The cytosol-associated iPLA_2_β participates in membrane phospholipid remodeling, signal transduction, cell proliferation, inflammation, and apoptosis [4,8,9]. Dysregulation of iPLA_2_β has been associated with several neurodegenerative, skeletal, and vascular smooth muscle, bone formation, and cardiac disorders [4,9]. Other reports reveal there is also iPLA_2_β induction in rodent models of T1D and human subjects with T1D [10,11,12].

Our examination of the link between iPLA_2_β and ER stress revealed that β-cells undergoing thapsigargin-induced ER stress have increased expression of iPLA_2_β and that inhibition of iPLA_2_β by *S*-bromoenol lactone (*S*-BEL), a select inhibitor of the β-isoform of iPLA_2_ [13], significantly attenuates β-cell apoptosis [5,14,15]. Consistent with a role for iPLA_2_β in this process, susceptibility to ER stress-induced apoptosis is amplified in INS-1 insulinoma cells overexpressing iPLA_2_β [5] or in islet β-cells from RIP-iPLA_2_β-Tg mice in which iPLA_2_β is selectively overexpressed only in the β-cells [15]. In contrast, the knockdown [16] or knockout [15] of iPLA_2_β reduced ER stress-induced β-cell apoptosis. Unexpectedly, in addition to its impact on iPLA_2_β, ER stress promoted accumulations in ceramides, not via the *de novo* pathway, but via the increased hydrolysis of sphingomyelins by neutral sphingomyelinase 2 (NSMase2) [17]. In turn, the ceramides served to trigger mitochondrial apoptotic pathways, resulting in β-cell apoptosis [18].

While eicosanoids have been the main focus in studies of β-cell death [19,20,21,22,23], the contribution of sphingolipids to this process are less-well characterized. Ceramides are complex pro-apoptotic lipids, and it has been long recognized that sphingomyelin-derived ceramides play important roles in decreasing cell proliferation and increasing apoptosis [24,25,26,27,28,29,30]. Recently, there has been a tremendous increase in interest in sphingolipid biology, and various sphingolipid derivatives have been identified as bioactive molecules [31,32] with important roles in cell signaling [33,34,35,36,37,38,39,40,41,42,43,44,45]. The intriguingly diverse and dynamic manner in which sphingolipids interact with one another [42] emphasizes not only how intricate the sphingolipid family is and how much still remains unknown but more importantly, the therapeutic potential of targeting the regulation of sphingolipid-generating pathways. Therefore, we considered the possibility of whether there were widespread changes in sphingolipids metabolism due to iPLA_2_β activation or due to ER stress in β-cells.

We used two β-cell models for our assessments: rat-derived INS-1 cells, genetically modified to overexpress iPLA_2_β, where ER stress was induced with thapsigargin, and mouse-derived Akita cells, which is a spontaneous model of ER stress. The Akita mouse, due to a mutation in *Ins2* gene, accumulates misfolded proinsulin in the ER, leading to the development of ER stress in β-cells. Prolonged ER stress promotes β-cell apoptosis, leading to hyperglycemia and diabetes in the Akita mouse within a few weeks after birth [46]. β-cell lines derived from the Akita mouse exhibit an analogous spontaneous ER stress and a higher incidence of apoptosis, relative to β-cells derived from wild-type (WT) mice [16]. Utilizing mass spectrometry protocols, we report for the first time that ER stress leads to iPLA_2_β-mediated differential distribution of sphingolipids, favoring the generation of pro- over anti-apoptotic sphingolipids, in β-cells. These data highlight a pivotal role for iPLA_2_β in modulating sphingolipids profile in β-cells, and as a consequence, their survival.

## 2. Results

### 2.1. Altered iPLA_2_β Expression, Chemical ER Stress Induction, and INS-1 Cell Sphingolipids

#### 2.1.1. Verification of the INS-1 Cell Model

**Figure 1:**
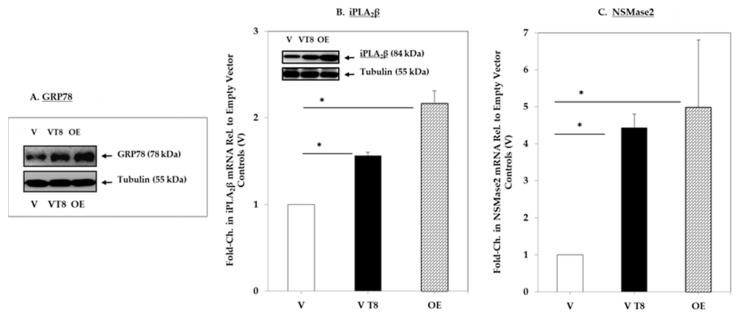
GRP78, iPLA_2_β, and NSMase2 expression in INS-1 cells in the absence and presence of thapsigargin (T). Protein and cDNA were prepared from empty vector (V) INS-1 cells treated with DMSO (V) or thapsigargin (1 µM for 8 h (VT8)) and iPLA_2_β-overexpressing (OE) INS-1 cells for immunoblotting and RT-qPCR analyses, respectively. Tubulin was used as a loading control in the protein analyses. (**A**,**B**). RT-qPCRs: 18S served as internal control, and fold-change in mRNA was calculated using the delta C_T_ method (ΔC_T_) and expressed relative to V controls: (**A**) ER stress marker GRP78, (**B**) iPLA_2_β mRNA (*inset*, iPLA_2_β protein); and (**C**) NSMase2 mRNA. Data are presented as mean ± SEM, *n* = 3 per group. (* Significantly different from V control, *p* < 0.05.).

Initially, it was important to validate the INS-1 model for subsequent studies and immunoblotting and RT-qPCR analyses were performed to achieve this. Empty vector (V) cells cultured in the absence or presence of thapsigargin (T) were compared with iPLA_2_β-overexpressing (OE) cells. Under basal conditions, the expression of GRP78 (Figure 1A), the master regulator of the unfolded protein response to ER stress [47], iPLA_2_β (Figure 1B) and NSMase (Figure 1C), was higher in the OE cells relative to V cells. Treatment with T led to the induction of all three in the V cells (VT), relative to untreated vector (V) cells. These findings confirm the (a) iPLA_2_β phenotype of V and OE cells, (b) expression of higher NSMase2 and ER stress induction in the iPLA_2_β OE cells, and (c) induction of ER stress, iPLA_2_β, and NSMase2 in V cells by thapsigargin under conditions to be used for the following sphingolipids analyses. Furthermore, the results reveal the comparable induction of ER marker GRP78 and NSMase2 enzyme by the chemical stressor (T) and overexpression of iPLA_2_β (in OΕ cells).

#### 2.1.2. Sphingolipids Profiles in INS-1 Cells

**Figure 2:**
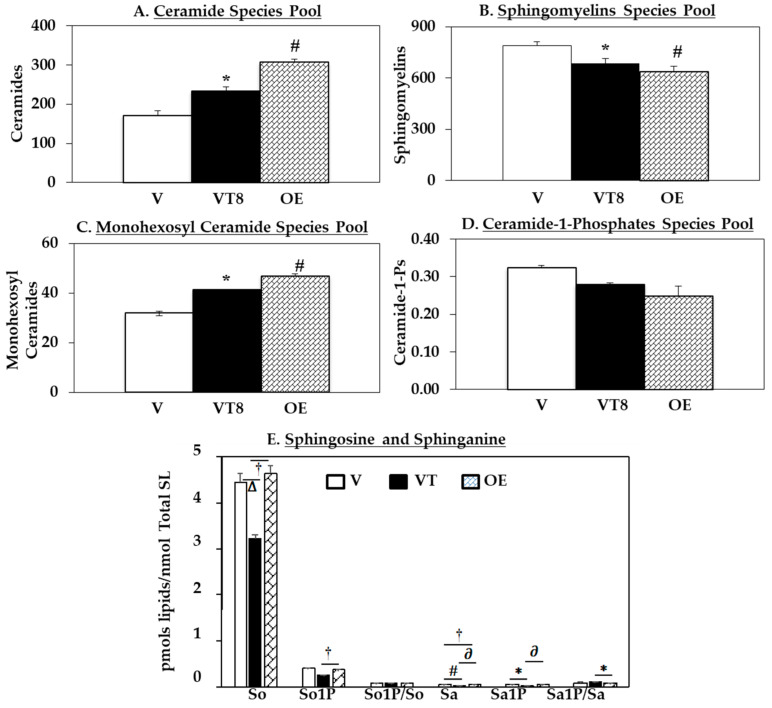
Sphingolipid (SL) abundances in INS-1 cells ± ER stress. Lipids were extracted from empty vector INS-1 cells treated with DMSO (V) or thapsigargin (T, 1 µM for 8 h, (VT8)) and iPLA_2_β-overexpressing (OE) INS-1 cells for LC ESI/MS/MS analyses: (**A**) Ceramide pool; (**B**) Sphingomyelin pool; (**C**) Monohexosyl ceramide pool; (**D**) Ceramide-1-phosphate pool; (**E**) Sphingosine and sphinganine. Data are presented as mean ± SEM pmols lipid/nmol total sphingolipid). One-Way ANOVA was utilized for statistical analysis. (**A**–**D**), * OE group significantly different from V and ^#^ VT group significantly different from V; *p* < 0.05; (**E**), * *p* < 0.05, **^†^** *p* < 0.01, **^∆^** *p* < 0.005, **^∂^** *p* < 0.0005, **^#^** *p* < 0.0001, *n* = 3 per group).

Next, LC-ESI/MS/MS analyses were used to assess the abundances of ceramide, sphingomyelin, monohexosyl ceramide (MHC), ceramide-1-phospate (C1P), and sphingosine (So) and sphingosine-1-phosphate (So1P) molecular species (Appendix A) under conditions that increase iPLA_2_β (OE and VT) cells compared to control (V) cells, as illustrated in Figure 2.

Ceramide molecular species were increased in the OE cells relative to V cells, as reflected by the higher pool of ceramides in the OE cells (Figure 2A). The induction of ER stress in V cells also resulted in an increase in the ceramide pool (in the VT cells) relative to untreated controls (V cells). As NSMase hydrolyzes sphingomyelins to generate ceramides, we investigated whether the observed increase in the ceramide pool was accompanied by changes in sphingomyelins. Indeed, we found similar decreases in the total pool of sphingomyelins (Figure 2B) in both models of increased ER stress and iPLA_2_β (OE and VT cells) relative to untreated controls (V cells).

Since ceramides can be converted to various sphingolipids that manifest opposite biological activity, we assessed their abundances in the various cell models. Following glycosylation, ceramides can form monohexosyl ceramides (MHCs) [48]. Here, we report an increase in the MHC pool in both the OE and VT groups relative to the V control cells (Figure 2C). Ceramides can also be phosphorylated to generate inflammatory C1P, which can activate cPLA_2_α and lead to the hydrolysis of arachidonic acid and PGE_2_ formation [49]. Similarly, it can be speculated that C1P activates iPLA_2_β to generate lipid mediators that serve to amplify β-cell apoptosis. However, we report that the C1P pools in OE and VT cells were not significantly different from the corresponding pool in the untreated V group (Figure 2D). Both non-phosphorylated and phosphorylated So and Sa sphingolipid species were decreased in the VT group relative to V (Figure 2E); however, they were all higher in the OE group relative to the VT group. Collectively, these findings suggest that in general, the increased expression of iPLA_2_β and NSMase2 (Figure 1) promotes alterations in the sphingolipids profile to favor the generation of lipids recognized to be detrimental and pro-apoptotic, and away from those that may be protective and anti-apoptotic.

### 2.2. Spontaneous ER Stress Induction and β-Cell Sphingolipids

#### 2.2.1. Verification of the Akita (AK) β-Cell Model

**Figure 3:**
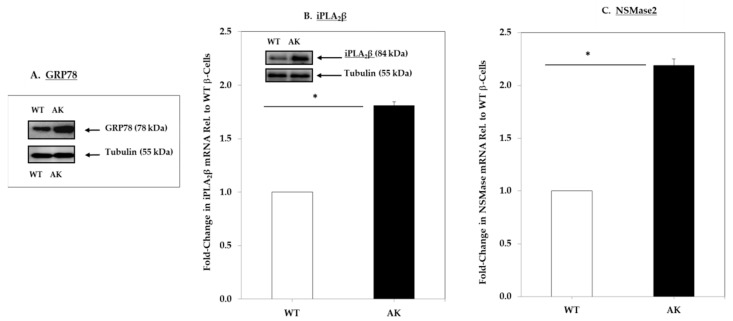
GRP78, iPLA_2_β, and NSMase expression in wild-type (WT) and Akita (AK) β-cells. Protein and cDNA were prepared from WT control and AK and analyzed as in Figure 1: (**A**) ER stress marker GRP78 protein, (**B**) iPLA_2_β message, and protein (*inset*); (**C**) NSMase2 message. (* Significantly different from WT control, *p* < 0.05). Data are presented as mean ± SEM, *n* = 3 per group.

Since the above observations were made in INS-1 cells that were genetically modified to express higher iPLA_2_β or chemically induced (thapsigargin, T) to exhibit ER stress, we sought to examine whether spontaneous ER stress in β-cells promotes similar changes in the sphingolipids profile. The Akita β-cells, due to a spontaneous mutation in the *Ins2* gene that leads to the generation and accumulation of pre-proinsulin in the ER, develop spontaneous ER stress [50,51]. We previously demonstrated that this occurs in the Akita β-cells without chemical intervention [52]. Here, immunoblotting and qRT-PCR analyses confirm the induction of GRP78 (Figure 3A), which is associated with the higher expression of both iPLA_2_β (Figure 3A) and NSMase2 (Figure 3C) in the AK, relative to wild-type (WT) cells. These analyses confirm that Akita cells, even under basal conditions, exhibit ER stress and that this is associated with higher iPLA_2_β and NSMase2.

#### 2.2.2. Sphingolipids Profiles in WT and Akita (AK) β-Cells

**Figure 4:**
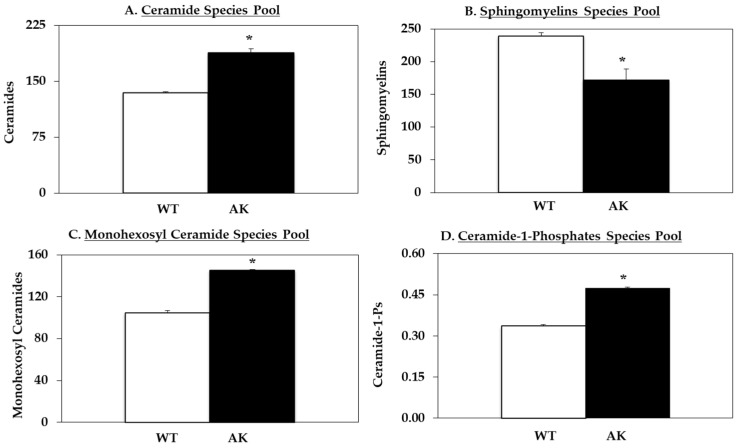
Basal sphingolipid (SL) abundances in WT and Akita β-cells. Lipids were extracted from empty WT and Akita β-cells for LC ESI/MS/MS analyses: (**A**) Ceramide pool; (**B**) Sphingomyelin pool; (**C**) Monohexosyl ceramide pool; (**D**) Ceramide-1-phosphate pool. Data are presented as mean ± SEM pmols lipid/nmol total sphingolipid). (* Significantly different from WT control, *p* < 0.05, *n* = 3 per group).

Next, LC-ESI/MS/MS analyses, similar to the ones used with INS-1 cells, were performed to assess the relative abundances of sphingolipids (Appendix A) in the WT and AK cells (Figure 4). We report that associated with the spontaneous ER stress and higher basal iPLA_2_β levels, we observed increases in the pool of ceramides in the AK cells, relative to WT cells (Figure 4A). In view of the higher basal NSMase2, we examined for changes in sphingomyelins and found that the pool of sphingomyelin molecular species was decreased in the AK cells, relative to WT cells (Figure 4B). Further, the pools of MHC molecular species (Figure 4C) and the pro-apoptotic/inflammatory [53] C1P species (Figure 4D) were higher in the AK cells relative to WT cells. In general, the qualitative changes in AK cells were analogous to those described above in OE and VT cells, relative to untreated controls (V cells), reinforcing the suggestion that both iPLA_2_β increases and ER stress modify the sphingolipids profile to favor pro-apoptotic species in β-cells.

#### 2.2.3. Effects of Inactivating iPLA_2_β on the Sphingolipids Profile in Akita (AK) β-Cells

**Figure 5:**
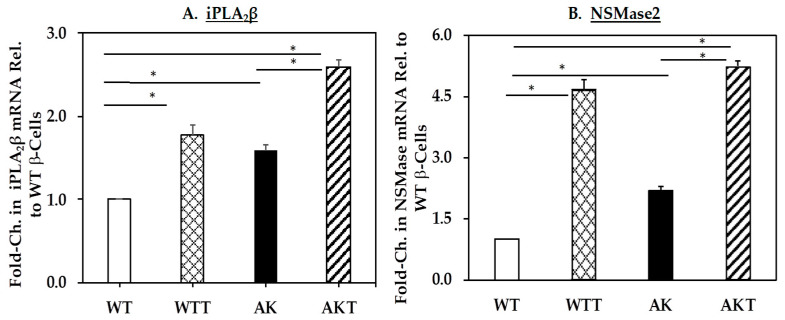
iPLA_2_β and NSMase expression ± thapsigargin. mRNA was determined and quantified as described in Figure 1 and expressed relative to WT controls: (**A**) iPLA_2_β mRNA; and (**B**) NSMase2 mRNA. (* Groups significantly different, *p* < 0.05) Data are presented as mean ± SEM, *n* = 3 per group.

As the changes in sphingolipids described in INS-1 and AK β-cells correlated with increased iPLA_2_β, we examined whether they could be reversed by the inactivation of iPLA_2_β. Further, because basal iPLA_2_β expression is higher in AK cells and they exhibit spontaneous ER stress, we examined the effects of *S*-BEL in AK cells in the absence and presence of thapsigargin (T). We find that both iPLA_2_β (Figure 5A) and NSMase2 (Figure 5B) are induced in the presence of thapsigargin (T), in both WTT and AKT cells, relative to WT and AK control cells, respectively.

**Figure 6:**
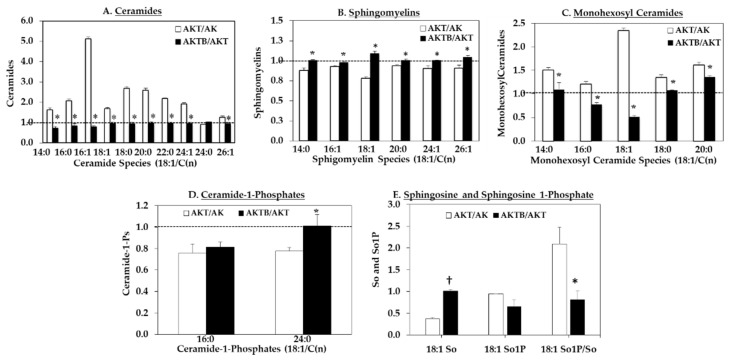
Effects of iPLA_2_β inhibition on sphingolipid abundances in Akita β-cells ± ER stress. Lipids were extracted from Akita β-cells treated with thapsigargin (T, 1 µM for 8 h) in the absence and presence of *S*-BEL (10 µM). The ratio of sphingolipids ± T (AKT/AT) and T ± *S*-BEL (AKTB/AKT) are presented as mean ± SEM pmols lipid/nmol total sphingolipid: (**A**) Ceramide pool; (**B**) Sphingomyelin pool; (**C**) Monohexosyl ceramide pool; (**D**) Ceramide-1-phosphate pool; and (**E**) Sphingosine (So) and So1P. Two-Way ANOVA was utilized for statistical analysis. (*^,†^ Significantly different from AKT/AK; *p* < 0.05 and *p* < 0.0005, *n* = 3 per group).

We next assessed the effects of chemical inhibition of iPLA_2_β by *S*-BEL on the sphingolipid profile in AK cells. The cells were pre-treated with *S*-BEL for 30 min prior to treatment with T. After 8 h, the cells were processed for sphingolipid analyses, as above, and the fold-change in sphingolipid species abundances in AKT cells relative to vehicle-treated cells (AK) was compared with those in AK cells treated with T + *S*-BEL (AKTB) relative to AKT. As expected, the ceramide molecular species were increased by thapsigargin (AKT/AK > 1, Figure 6A), but this effect was reversed by *S*-BEL (AKTB/AKT ≤ 1) in all species except 18:1/24:0. Concurrently, thapsigargin-induced decreases in sphingomyelin molecular species (AKT/AK < 1, Figure 6B) were reversed by *S*-BEL. Similarly, various MHC species increased with thapsigargin (Figure 6C) were also decreased and reversed by *S*-BEL (AKTB/AKT ≥ 1). However, the C1P species exhibited a differential regulation, where two species (18:1/16:0 and 18:1/24:0) were modestly decreased following thapsigargin treatment (AKT/AK > 1) but only 18:1/24:0 was rescued by *S*-BEL (AKTB/AKT > 1) (Figure 6D). While no differences were evident in Sa or Sa1P among the groups, so was increased in the AK, relative to AKT, and the ratio of So1P/So decreased with *S*-BEL (AKTB).

## 3. Discussion

It has long been established that sphingolipids play important and dynamic roles in cellular physiology and that their levels are maintained under tightly regulated homoeostasis processes via a variety of highly specialized enzymes [54,55,56,57]. We previously demonstrated that ER stress-mediated β-cell apoptosis is mediated, in part, through the activation of iPLA_2_β and that an increased expression of iPLA_2_β amplifies the susceptibility of β-cells to ER stress and subsequent β-cell apoptosis [4,5,8,16,17]. Increases in iPLA_2_β and ER stress were also associated with accumulations in ceramides, which contributed to β-cell death by triggering the intrinsic apoptotic pathway [4,18]. These observations provided evidence for the involvement of an iPLA_2_β/ceramide axis in promoting β-cell dysfunction and apoptosis, which are key contributors to the development of diabetes [1,2]. As reports from various experimental models and clinical settings suggested that ER stress contributes to β-cell apoptosis during the evolution of diabetes [4], we set out to assess the link between ER stress, iPLA_2_β, and β-cell sphingolipids in the present study.

The roles of ceramides are related to their involvement in cell survival and are mainly considered to be pro-apoptotic [58,59,60,61]. Ceramide generation can occur through multiple pathways: (a) *de novo*, initiated by the condensation of serine and palmitoyl CoA, which is catalyzed by the rate-limiting enzyme serine palmitoyl transferase (SPT) [62]; (b) sphingomyelin hydrolysis by sphingomyelinases [48,63]; or (c) salvage pathway involving ceramidase [64]. Ceramides can be further converted to C1P by ceramide kinase or to MHCs by glucosylceramide synthase or ceramide galactosyl transferase [48]. Our work [14,15,16,17,18] indicates that the accumulation of ceramides during β-cell apoptosis due to ER stress occurs through the hydrolysis of sphingomyelins by NSMase2. Therefore, we aimed at determining whether the overall sphingolipid profile is modulated by iPLA_2_β in β-cells undergoing ER stress.

In the present study, MS protocols were applied to INS-1 cells and Akita β-cells to discern the impact of ER stress and iPLA_2_β expression on the abundances of various sphingolipids. The INS-1 cells are widely used in studies of β-cell function and survival and the availability in our laboratory of genetically modified iPLA_2_β-overexpressing (OE) INS-1 cells permitted examination of the effects of higher iPLA_2_β expression in the absence of chemically induced ER stress. In addition, parallel comparisons with thapsigargin-treated empty-vector (V) INS-1 cells enabled assessment of the effects of ER stress induction in the absence of genetic modification of iPLA_2_β expression. The Akita β-cells (AK) provided a model of spontaneous ER stress that also expresses higher basal iPLA_2_β [16], thus precluding consideration of the effects of chemical intervention or genetic modification.

Prior to the lipid analyses, immunoblotting and RT-PCR analyses confirmed (a) increased basal expression of iPLA_2_β, NSmase2, and GRP78 in the OE and AK cells, relative to V and WT cells, respectively and (b) induction of ER stress, iPLA_2_β, NSMase2, and GRP78 following exposure to thapsigargin in VT and AKT cells, relative to V and AK cells, respectively. Subsequently, LC-ESI/MS/MS analyses were used to identify molecular species of ceramides, sphingomyelins, monohexosyl ceramides (MHCs), ceramide-1-phosphate (C1P), sphinganine (Sa), sphingosine (So), and the So phosphate derivatives So1P.

Under basal conditions, comparisons of OE vs. V cells reveal that higher iPLA_2_β expression leads to (a) increases in the pro-apoptotic [65] ceramides, (b) decreases in anti-apoptotic [17] sphingomyelins, (c) increases in the primarily apoptotic [48] MHCs, (d) no change in inflammatory and apoptotic [66] C1P, and (e) no change in anti-apoptotic [53] So1P. Comparisons of VT vs. V cells following induction of ER stress, as reflected by increased expression of ER stress marker GRP78, and of iPLA_2_β in the VT cells revealed changes in VT sphingolipids that were similar to those in the OE cells.

Similar comparisons between the WT and AK cells under basal conditions revealed relative changes in the AK cells that mirrored those in the OE cells including (a) increases in ceramides, (b) decreases in sphingomyelins, (c) and increases in the MHCs. However, differences were noted in the C1P molecular species (unchanged in OE but increased in AK) and in the So1P/So ratio (unchanged in OE but increased in AKT). This is an important observation, as we also noted an increase in So1P/So ratio in the non-obese diabetic (NOD) mice, an autoimmune model of T1D [67]. The NOD mice develop insulitis at 4–6 weeks of age and spontaneous T1D starting at 16–18 weeks of age [68]. β-Cell death and the development of T1D in the NOD is preceded by the induction of ER stress in the β-cells [69,70]. Given that So1P has been identified as a lipid that promotes T-cell migration and retention in inflamed tissues [71], it is likely that it could manifest deleterious effects in β-cells also.

Exposure to the ER stressor, thapsigargin, amplified the changes in the sphingolipids in AK cells, suggesting that ER stress and consequential downstream processes impact the sphingolipids profile. Since iPLA_2_β is also further induced, we considered the possibility that its activation contributes to the observed changes in sphingolipids. To assess this, AK cells were pre-treated with *S*-BEL prior to thapsigargin exposure, and this was found to reverse nearly all changes in the sphingolipids, with the exception of d18:1/16:0 C1P, toward control abundances. These findings are consistent with the involvement of iPLA_2_β activation in the regulation of β-cell sphingolipids.

**Figure 7:**
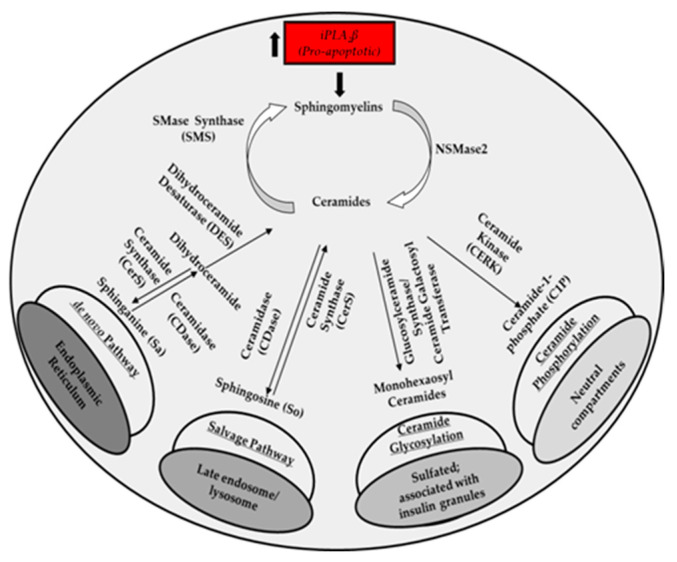
Proposed model of the impact of iPLA_2_β activation of β-cell sphingolipids. Ceramides are the central species, and arrows in brackets indicate changes to the specific lipid-derived species in conditions that increase iPLA_2_β and in turn favor pro-apoptotic outcomes.

While our data demonstrate similar modifications in the sphingolipids profile with higher iPLA_2_β and ER stress, differences were noted with respect to specific molecular species that were affected. Such discrepancies are not unexpected considering the inherent differences between the models studied, which include the (a) the high basal but long-term expression of iPLA_2_β in the iPLA_2_β-overexpressing INS-1 cells, which promotes ER stress vs. (b) the transient, short-term, chemically induced (thapsigargin) ER stress, which promotes iPLA_2_β expression in the Vector INS-1 cells vs. (c) the basal spontaneous and prolonged ER stress, which is associated with higher iPLA_2_β expression in the AK cells. In view of these differences, it is plausible that the sphingolipids-generating pathways are regulated dissimilarly, giving rise to variations in specific molecular species changes. Furthermore, recent reports suggest that the fate of different sphingolipids and activities of their converting enzymes [72,73,74,75] may be cell-specific and even compartment-specific [31,48]. As reviewed extensively elsewhere [34,48,72,76,77,78,79,80,81], the sphingolipids-generating pathways are quite complex, involving inter-conversion between the sphingolipids and occurring in multiple subcellular organelles. In this regard, while iPLA_2_β is localized in the cytosol under basal conditions, ER stress causes a temporal re-distribution of iPLA_2_β to the ER, the mitochondria, and the nucleus [5,82]. The compartmentalized and subcellular localization (i.e., endosome/lysosome, endoplasmic reticulum, secretory granules; see Figure 7) of different pools of sphingolipids [48] raises the likelihood that the sphingolipids in different subcellular organelles could be affected at different rates depending on the duration of ER stress and/or iPLA_2_β induction and subcellular mobilization.

Although our studies provided evidence of significant changes in sphingolipids associated with altered iPLA_2_β expression and the development of ER stress, several questions remain to be addressed. These include the following: (1) identification of the hexosyl component of MHCs; because this was not discernible from the present analyses, it will be important to determine if the species increased are apoptotic glucosyl-ceramides, (2) in addition to NSMase, what and how are the other enzymes of the sphingolipids generating pathways altered by iPLA_2_β activation and/or ER stress, (3) which molecular species of the various sphingolipids are most prone to be affected and influenced by duration of elevated iPLA_2_β or ER stress, (4) what specific sphingolipid changes correlate with β-cell apoptosis, and importantly (5) whether alterations in sphingolipids contribute to β-cell apoptosis in the context of diabetes development These labor-intensive issues and other questions of the involved underlying mechanisms are currently being addressed in our laboratory utilizing autoimmune rodent models of type 1 diabetes.

## 4. Materials and Methods

### 4.1. Materials

Materials and (sources) were as follows: Akita (AK) and wild-type (WT) β-cells were generously provided by Dr. Akio Koizumi (Kyoto Graduate School of Medicine, Kyoto, Japan); Ultra-Performance Liquid Chromatography was a Shimadzu Nexera 30-AD system, and the mass spectrometer was a 5500 QTRAP from ABSciex (Applied Biosystems, MDS Sciex); internal standards for MS analyses (Avanti Polar Lipids, Alabaster, AL, USA); *S*-BEL (Cayman Chemicals, Ann Arbor, MI, USA); cell lifters (Fisher Healthcare, ThermoFisher Scientific, Houston, TX, USA); Superscript III First-strand Synthesis System and Fast SYBR^®^ Green PCR Master Mix, Roswell Park Memorial Institute (RPMI) 1640 medium, Dulbecco’s modified Eagle’s (DMEM) medium (Life Technologies Corporation, Grand Island, NY, USA); RNeasy kit (Qiagen Inc, Valencia, CA, USA); Group VIA iPLA_2_β antibody T-14 (sc-14463), GRP78 (sc-1050), β-actin (sc-47778), and α-tubulin TU-02 (sc-8035) primary antibodies (Santa Cruz Biotechnology, Inc., Santa Cruz, CA, USA); thapsigargin (Sigma Co., St. Louis, MO, USA); LC/MS grade water, methanol and chloroform, formic acid and ammonium formation were purchased from (ThermoFisher-Scientific, Waltham, MA, USA); Phenomenex Kinetex 2.6 μm C18 100A 50 × 2.1 mm reverse phase HPLC chromatography column (Torrance, CA, USA); and disposable borosilicate glass culture tubes with PTFE-lined caps (VWR Laboratory Products, Westbury, NY, USA).

### 4.2. INS-1 Cell Culturing and Treatment

INS-1 (empty vector or iPLA_2_β-overexpressing (OE)) cells were generated and cultured, as described [5]. Briefly, cells were cultured in RPMI 1640 medium, containing 11 mM glucose, 10% fetal calf serum, 10 mM HEPES buffer, 2 mM glutamine, 1 mM sodium pyruvate, 50 mM mercaptoethanol (BME), and 0.1% (*w/v*) each of penicillin and streptomycin in cell culture conditions (37 °C, 5%CO_2_/95% air). Mouse Akita and WT β-cells were generated and cultured, as described [16,52,83]. Briefly, cells were cultured in DMEM medium containing 15% fetal calf serum, β-mercaptoethanol (150 µM), and 0.1% (*w/v*) each of penicillin and streptomycin in cell culture conditions (37 °C, 5%CO_2_/95% air). For both cell lines, the medium was changed every other day, and the cells were passaged once a week. Thapsigargin (T) was prepared in DMSO and used at a final concentration of 1 μM. In some experiments, the cells were pre-treated for 30 min with the iPLA_2_β inhibitor, *S*-BEL (10 µM). Then, the cells were washed prior to treatment with vehicle or thapsigargin. All experimental protocols included DMSO vehicle-only treated triplicate controls.

### 4.3. Quantitative Real-Time PCR

INS-1 cells (empty-Vector and OE) and WT and Akita β-cells were treated with DMSO alone or thapsigargin (1 µM) for 8 h. Total RNA was prepared using the RNeasy kit, and double-stranded cDNA was generated using the Superscript III First-strand Synthesis System kit, as described [14,15,17,18]. Real-time PCR was performed using Fast SYBR^®^ Green PCR Master Mix and carried out in a plate-based LightCycler^®^ 480 System (Roche Life Sciences). The primers were designed based on known sequences for iPLA_2_β, NSMase2, and 18S, and the respective Gene Bank Gene ID numbers are as follows; Rat: #360426, #83537, and #100861533 and Mouse: #53357, #20598, and #18519791. Primer sets (sense/antisense) were as follows; Rat: iPLA_2_β, tgtgacgtggacagcactagc/ccccagaaacgactatgga; NSMase, ccggatgcacactacttcagaa/ggattgggtgtctggagaaca; and 18S, agtcctgccctttgtacaca/gatccgagggcctcactaaac and Mouse: iPLA_2_β, agcttcaattcatgcagttctttggacgc/ttcgatatcgggagatagcagcagctgg; NSMase, ccggatg-cacactacttcagaa/ggattgggtgtctggagaaca; and 18S, agtcctgccctttgtacaca/gatccgagggcctcactaaac.

### 4.4. Immunoblotting

Cells were harvested following treatment and prepared for SDS-PAGE analyses, as described [5]. Briefly, harvested cells were washed once with PBS, sonicated, protein concentration was determined by the Bradford Protein Assay, and 30 μg of protein were analyzed on a 10% acrylamide SDS-PAGE gel. Resolved proteins were transferred onto Immobilon-P polyvinylidene difluoride (PVDF) membranes and probed (1°/2°) for iPLA_2_β (1:500/1:1000), GRP78 (1:250/1:1000), and loading controls tubulin and actin both at (1:500/1:1000). Bands of interest were visualized by Universal Hood II Gel Imager (Bio-Rad) and densitometry analyses were carried out using the accompanying Image Lab software.

### 4.5. LC-Electrospray Ionization (ESI)-MS/MS Analyses of Sphingolipids

Cells were plated separately in 10 cm cell culture dishes to provide approximately 1 × 10^6^ cells at harvesting. Following an 8 h treatment period with DMSO vehicle or thapsigargin, the cell culture dishes were placed on ice, washed twice with phosphate-buffered saline (PBS), and harvested by scraping in 200 μL of PBS. The cells were immediately frozen and stored at −80 °C. At the time of analyses, the cells were thawed on ice followed by sonication to obtain a homogenous mixture, and lipids were extracted using a modified Bligh and Dyer method and analyzed, as described [84]. Briefly, to 200 μL of the cells in PBS, 1.5 mL of 2:1 methanol/chloroform was added. The samples were spiked with internal standards consisting of 50 pmol each of d17:1 sphingosine, d17:0 sphinganine, d17:1 sphingosine-1-phosphate, d17:0 sphinganine-1-phosphate, d18:1/12:0 ceramide-1-phosphate, d18:1/12:0 sphingomyelin, d18:1/12:0 ceramide, and d18:1/12:0 monohexosyl ceramide. The mixture thus obtained was sonicated to disperse the cell clumps and incubated for 6 h at 48 °C. Following incubation, the samples were sonicated, followed by centrifugation to separate particulates. Then, the extracts were dried under vacuum and reconstituted by sonicating in 500 μL of methanol followed by incubation at 48 °C for 15 min, vortexing, and incubation for an additional 15 min at 48 °C. Then, the samples were centrifuged to separate particulates, and 10 μL was used for analysis. The lipids were separated using a Kinetix C18 column (50 × 2.1 mm, 2.6 μm) on a Nexera ultra-high-performance liquid chromatography system and eluted using a linear gradient (solvent A, 58:41:1 CH_3_OH/water/HCOOH 5 mM ammonium formate; solvent B, 99:1 CH_3_OH/HCOOH 5 mM ammonium formate, 20–100% B in 3.5 min and at 100% B for 4.5 min at a flow rate of 0.4 mL/min at 60 °C). Electrospray ionization (ESI) with tandem mass spectroscopy (MS/MS) on a 5500 QTRAP instrument was utilized for the detection and quantitation of analytes in the positive ion mode. Multiple reaction monitoring transitions utilized for the analytes are listed in Appendix A.

### 4.6. Statistical Analyses

Data were converted to mean ± standard error of the means (SEMs), Student’s *t*-test was applied to determine significant differences (*p* < 0.05) between two samples, and One-Way and Two-Way ANOVA statistical analyses were applied to determine significant differences (at *p* < 0.05) between more than two sample groups.

## 5. Conclusions

In summary, our data demonstrate for the first time that iPLA_2_β and ER stress in β-cells, in concert, modulate the sphingolipids profile in β-cells and that the consequential redistribution between the various sphingolipids favors apoptosis over the survival of the β-cells. To our knowledge, molecular species assessments of ceramide-derived sphingolipids have not been examined in detail in β-cells, and our findings provide the basis for future studies that will address important issues in a cell system where the dysregulation of sphingolipids generation could impact its survival, and as a consequence, could play a critical role in the evolution of diabetes or other inflammatory diseases [85,86].

## Data Availability

Not applicable.

## Abbreviations

AK, Akita β-cells; C1P, ceramide-1-phospahate; CM, ceramide; iPLA_2_β, cytosol-associated Ca^2+^-independent phospholipase A_2_; MHC, monohexosyl ceramide; OE, iPLA_2_β-overexpressing INS-1 cells; NOD, non-obese diabetic, NSMase2, neutral sphingomyelinase 2; Sa, sphinganine; Sa1P, sphinganine-1-phosphate; SM, sphingomyelin; So, sphingosine; So1P, sphingosine-1-phosphate; PTFE, polytetrafluoroethylene; V, empty-vector INS-1 cells; and WT, wild-type β-cells.

## Appendix A

**Table A1:** MRM Transitions monitored for the investigated sphingolipid species.

Species	Precursor Ion (*m/z*)	Product Ion (*m/z*)	DP	CE
d_17:1_ Sphingosine	286.4	268.3	120	15.0
d_17:0_ Sphinganine	288.4	270.4	120	21.0
d_18:1_ Sphingosine	300.5	282.3	120	21.0
d_18:0_ Sphinganine	302.5	284.3	120	21.0
d_17:1_ S1P	366.4	250.4	120	23.0
d_17:0_ Sa1P	368.4	252.4	120	23.0
d_18:1_ S1P	380.4	264.4	120	25.0
d_18:0_ Sa1P	382.4	266.4	120	25.0
d_18:1/12:0_ Ceramide	482.6	264.4	80.0	41.0
d_18:1/12:0_ C1P	562.4	264.4	80.0	41.0
d_18:1/12:0_ MonoHex	644.6	264.4	80.0	41.0
d_18:1/12:0_ SM	647.7	184.4	80.0	41.0
d_18:1/14:0_ Ceramide	510.7	264.4	80.0	43.5
d_18:1/14:0_ C1P	590.4	264.4	80.0	43.5
d_18:1/14:0_ MonoHex	672.6	264.4	80.0	43.5
d_18:1/14:0_ SM	675.7	184.4	80.0	43.5
d_18:1/16:0_ Ceramide	538.7	264.4	80.0	46.0
d_18:1/16:0_ C1P	618.5	264.4	80.0	46.0
d_18:1/16:0_ MonoHex	700.7	264.4	80.0	46.0
d_18:1/16:0_ SM	703.8	184.4	80.0	46.0
d_18:0/16:0_ Ceramide	540.7	266.4	80.0	46.0
d_18:0/16:0_ C1P	620.5	266.4	80.0	46.0
d_18:0/16:0_ MonoHex	702.5	266.4	80.0	46.0
d_18:0/16:0_ SM	705.8	184.4	80.0	46.0
d_18:1/18:1_ Ceramide	564.7	264.4	80.0	48.5
d_18:1/18:1_ C1P	644.5	264.4	80.0	48.5
d_18:1/18:1_ MonoHex	728.7	264.4	80.0	48.5
d_18:1/18:1_ SM	731.8	184.4	80.0	48.5
d_18:1/18:0_ Ceramide	566.7	264.4	80.0	48.5
d_18:1/18:0_ C1P	646.5	264.4	80.0	48.5
d_18:1/18:0_ MonoHex	730.7	264.4	80.0	48.5
d_18:1/18:0_ SM	733.8	184.4	80.0	48.5
d_18:1/20:0_ Ceramide	594.7	264.4	80.0	51.0
d_18:1/20:0_ C1P	674.4	264.4	80.0	51.0
d_18:1/20:0_ MonoHex	756.7	264.4	80.0	51.0
d_18:1/20:0_ SM	759.9	184.4	80.0	51.0
d_18:1/22:0_ Ceramide	622.8	264.4	80.0	53.5
d_18:1/22:0_ C1P	702.7	264.4	80.0	53.5
d_18:1/22:0_ MonoHex	784.8	264.4	80.0	53.5
d_18:1/22:0_ SM	787.9	184.4	80.0	53.5
d_18:1/24:1_ Ceramide	648.9	264.4	80.0	56.0
d_18:1/24:1_ C1P	728.6	264.4	80.0	56.0
d_18:1/24:1_ MonoHex	810.9	264.4	80.0	56.0
d_18:1/24:1_ SM	813.9	184.4	80.0	56.0
d_18:1/24:0_ Ceramide	650.9	264.4	80.0	56.0
d_18:1/24:0_ C1P	730.6	264.4	80.0	56.0
d_18:1/24:0_ MonoHex	812.9	264.4	80.0	56.0
d_18:1/24:0_ SM	815.9	184.4	80.0	56.0
d_18:1/26:1_ Ceramide	676.9	264.4	80.0	58.5
d_18:1/26:1_ C1P	756.7	264.4	80.0	58.5
d_18:1/26:1_ MonoHex	838.9	264.4	80.0	58.5
d_18:1/26:1_ SM	841.9	184.4	80.0	58.5
d_18:1/26:0_ Ceramide	678.9	264.4	80.0	58.5
d_18:1/26:0_ C1P	758.7	264.4	80.0	58.5
d_18:1/26:0_ MonoHex	840.9	264.4	80.0	58.5
d_18:1/26:0_ SM	843.9	184.4	80.0	58.5

SCIEX 5500 QTRAP mass spectrometer settings for reverse phase chromatographic separation of various sphingolipids. DP: declustering potential, CE: collision energy, EP: entrance potential, CXP: cell exit potential. Transitions and settings were determined manually via direct infusion. Settings and transitions were chosen based on the best signal obtained during infusion using manual tuning in SCIEX Analyst software.

**Table A2:** INS-1 cell sphingolipid molecular species.

**PANEL A**												
**SL** **Molecular Species**	**Ceramides**						**Sphingomyelins**					
	**V**		**OE**		**VT**		**V**		**OE**		**VT**	
	**Mean**	**SE**	**Mean**	**SE**	**Mean**	**SE**	**Mean**	**SE**	**Mean**	**SE**	**Mean**	**SE**
**d18:1/14:0**	1.04	0.07	* 1.44	0.10	1.24	0.10	13.93	1.29	* 19.63	0.43	11.30	0.54
**d18:1/16:0**	18.23	2.51	26.21	2.40	^∆^ 35.82	0.87	119.04	3.36	^∆^ 67.29	5.69	^∆^ 89.11	3.49
**d18:0/16:0**	0.46	0.08	0.63	0.07	0.64	0.05	0.77	0.08	0.84	0.16	0.56	0.04
**d18:1/18:1**	0.30	0.02	0.32	0.02	^#^ 1.18	0.03	36.50	1.34	31.23	1.99	31.56	1.56
**d18:1/18:0**	8.99	1.09	8.76	0.84	^∆^ 26.82	0.80	117.10	2.42	* 101.91	2.53	^∆^ 98.12	1.98
**d18:1/20:0**	1.08	0.11	1.18	0.08	^∆^ 2.86	0.11	76.08	4.21	65.69	2.97	63.87	3.24
**d18:1/22:0**	17.42	1.36	^†^ 27.82	1.53	^∆^ 53.23	2.35	100.50	2.34	^†^ 117.89	2.49	93.25	3.67
**d18:1/24:1**	15.34	1.48	* 23.30	1.67	^∆^ 44.28	2.20	94.46	0.70	^†^ 115.67	1.36	81.20	5.71
**d18:1/24:0**	105.88	4.74	^∆^ 138.96	3.71	^†^ 135.46	2.70	221.16	5.03	^∆^ 154.23	9.54	^†^ 151.16	13.93
**d18:1/26:1**	0.42	0.02	^∆^ 0.68	0.03	^†^ 0.75	0.05	3.66	0.53	4.33	0.29	6.35	0.97
**d18:1/26:0**	2.81	0.10	^∂^ 4.75	0.11	^†^ 4.74	0.30	6.45	0.42	^∆^ 9.34	0.05	7.95	0.49
**PANEL B**												
**SL Molecular Species**	**Monohexosyl Ceramides**						**Ceramide-1-Phosphates**					
	**V**		**OE**		**VT**		**V**		**OE**		**VT**	
	**Mean**	**SE**	**Mean**	**SE**	**Mean**	**SE**	**Mean**	**SE**	**Mean**	**SE**	**Mean**	**SE**
**d18:1/14:0**	0.05	0.00	0.06	0.01	^∂^ 0.08	0.00	0.03866	0.01027	0.04127	0.00461	0.03362	0.00457
**d18:1/16:0**	4.23	0.07	^∆^ 4.97	0.06	^∂^ 9.84	0.51	0.23508	0.03697	0.19329	0.04573	0.17544	0.04935
**d18:0/16:0**	1.85	0.16	^∆^ 4.33	0.27	2.11	0.09	0.00393	0.00287	0.00113	0.00036	0.00308	0.00099
**d18:1/18:1**	0.07	0.01	* 0.05	0.00	^∂^ 0.22	0.01	0.19495	0.07664	0.11587	0.06149	0.20923	0.13018
**d18:1/18:0**	1.86	0.08	^∆^ 1.21	0.05	^∂^ 3.83	0.11	0.05083	0.00469	0.05130	0.00176	0.04164	0.01235
**d18:1/20:0**	0.14	0.00	^∆^ 0.11	0.00	^#^ 0.26	0.00	0.00907	0.00317	0.00927	0.00361	0.00835	0.00351
**d18:1/22:0**	2.96	0.10	3.08	0.06	^∂^ 4.48	0.11	0.03667	0.01250	0.04694	0.00929	0.02706	0.00633
**d18:1/24:1**	1.26	0.14	1.49	0.02	^#^ 2.74	0.06	0.04243	0.01041	0.04558	0.00501	0.04003	0.00845
**d18:1/24:0**	25.29	0.73	^∆^ 31.14	0.36	^∆^ 34.12	0.82	0.30555	0.03705	0.57806	0.01633	0.23822	0.02777
**d18:1/26:1**	0.10	0.01	0.16	0.03	0.11	0.00	0.01189	0.00665	0.01646	0.00444	0.01715	0.00547
**d18:1/26:0**	0.56	0.02	^∆^ 0.81	0.01	* 0.76	0.05	0.00721	0.00205	* 0.01152	0.00117	0.00902	0.00285

INS-1 cell sphingolipid molecular species. Mass spectrometry techniques were utilized to decipher ceramide species in INS-1 cells that contained empty iPLA_2_β vector control cells (V), ER stress inducer thapsigargin (T) treated V cells (VT) and iPLA_2_β-overexpressing cells (OEs). Ceramide species investigated included ceramides and sphingomyelins **(PANEL A)** and monohexosyl ceramide and ceramide-1-phosphates **(PANEL B)**. Data are represented as SEM ± SE (*n* = 3 per group) with varied statistical significance from control V cells; * *p* < 0.05, ^†^ *p* < 0.01, ^∆^ *p* < 0.005, ^∂^ *p* < 0.0005, ^#^ *p* <0.0001.
